# Residency, Habitat Use and Sexual Segregation of White Sharks, *Carcharodon carcharias* in False Bay, South Africa

**DOI:** 10.1371/journal.pone.0055048

**Published:** 2013-01-28

**Authors:** Alison Kock, M. Justin O’Riain, Katya Mauff, Michael Meÿer, Deon Kotze, Charles Griffiths

**Affiliations:** 1 Department of Zoology, University of Cape Town, Cape Town, South Africa; 2 Shark Spotters, Cape Town, South Africa; 3 Department of Statistical Sciences, University of Cape Town, Cape Town, South Africa; 4 Department of Environmental Affairs, Oceans and Coasts Branch, Cape Town, South Africa; University of California Davis, United States of America

## Abstract

White sharks (*Carcharodon carcharias*) are threatened apex predators and identification of their critical habitats and how these are used are essential to ensuring improved local and ultimately global white shark protection. In this study we investigated habitat use by white sharks in False Bay, South Africa, using acoustic telemetry. 56 sharks (39 female, 17 male), ranging in size from 1.7–5 m TL, were tagged with acoustic transmitters and monitored on an array of 30 receivers for 975 days. To investigate the effects of season, sex and size on habitat use we used a generalized linear mixed effects model. Tagged sharks were detected in the Bay in all months and across all years, but their use of the Bay varied significantly with the season and the sex of the shark. In autumn and winter males and females aggregated around the Cape fur seal colony at Seal Island, where they fed predominantly on young of the year seals. In spring and summer there was marked sexual segregation, with females frequenting the Inshore areas and males seldom being detected. The shift from the Island in autumn and winter to the Inshore region in spring and summer by females mirrors the seasonal peak in abundance of juvenile seals and of migratory teleost and elasmobranch species respectively. This study provides the first evidence of sexual segregation at a fine spatial scale and demonstrates that sexual segregation in white sharks is not restricted to adults, but is apparent for juveniles and sub-adults too. Overall, the results confirm False Bay as a critical area for white shark conservation as both sexes, across a range of sizes, frequent the Bay on an annual basis. The finding that female sharks aggregate in the Inshore regions when recreational use peaks highlights the need for ongoing shark-human conflict mitigation strategies.

## Introduction

The depletion of top marine predators, particularly sharks, is of great concern, because their loss carries risks of ecosystem degradation [1], [2]. Sharks are highly susceptible to a range of anthropogenic influences [3], [4], [5] due to their life-history characteristics, including low fecundity, slow growth and late age of sexual maturity [6], [7], [8]. Furthermore, because many shark species are wide-ranging their effective protection necessitates a coordinated, global conservation effort including all areas that are critical for the different life history stages [9], [10].

White sharks *Carcharodon carcharias* are vulnerable to human impacts as they share all of the life history traits that threaten other shark species in addition to being apex predators with low abundance and circumglobal ranging patterns [11], [12]. Worldwide they are protected by CITES Appendix II, which restricts exploitation, and they are listed as “Vulnerable” by the IUCN [12]. They are protected in seven countries, including South Africa, but despite enacting protective legislation, there is limited knowledge available on how best to make such protection effective. Key to this objective is the identification of critical areas that function as nursery, breeding and feeding grounds and how the use of such areas varies in time and with the age and sex of individuals.

Whilst white sharks are known to engage in broad-scale coastal [13], [14], [15] and oceanic migrations [16], [13], [17], [18], [19], [20] they typically aggregate in select coastal areas [21], [22],[23],[24],[25],[15]. Sharks that frequent coastal regions are particularly vulnerable, as they are threatened by diverse anthropogenic activities including intensive shore-based fishing, pollution and the transformation or disturbance of natural habitat [26], [27], [9]. In South Africa, white sharks are most often associated with near shore, Cape fur seal (*Arctocephalus pusillus pusillus*) colonies in the Southern and Western Cape, but they have also been shown to frequent the inshore regions of False Bay [28], Mossel Bay [29] and KwaZulu-Natal [30]. Limited information is available on the extent and reason(s) for white sharks aggregating in inshore areas devoid of seal colonies. It is further not known whether there are differences in the sex and/or age classes of sharks frequenting inshore or island aggregation sites and whether these patterns vary seasonally.

Intersexual and size differences in migratory and aggregation patterns have been identified for white sharks, including differences in migration between the sexes for adult sharks off the coast of California [31], [14], Guadalupe Island, Mexico [24], Neptune Islands, Australia [32] and in the offshore area in the North Pacific [14], [33]. These studies report differences in the arrival and departure time of male and female sharks at aggregation sites, with females typically arriving and leaving earlier than males at the Neptune Islands, Australia, while males arrive and leave earlier than females in the Pacific. Furthermore, previous research has suggested a clear size-based preference for different prey species with white sharks ≤3 m feeding predominantly on teleosts and elasmobranchs, while white sharks >3 m supplement their diet with marine mammals, such as seals [18], [34], [35]. Thus we predict that there may be differences in behavioural patterns for white sharks at aggregation sites. There is limited data on the fine-scale habitat use and movement patterns of white sharks at aggregation sites in South Africa. In this study we use acoustic telemetry to test the null hypothesis that there is no sexual, size or seasonal differences in white shark residency and habitat use at a pinniped colony and the Inshore region of False Bay, South Africa.

## Methods

### Ethics Statement

Data were collected according to protocols approved by the University of Cape Town and South African Department of Environmental Affairs: Oceans and Coasts, and adhered to the legal requirements of South Africa. All research methods were approved and conducted under the South African Department of Environmental Affairs: Oceans and Coasts permitting authority. Permit # V1/1/5/1, V1/8/5/1.

### Study Site

**Figure 1:**
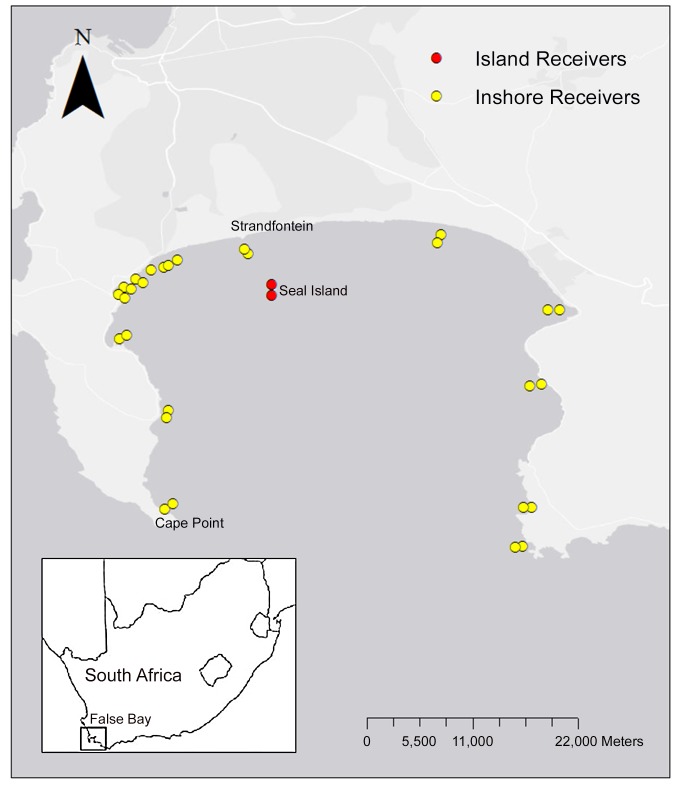
Locations of acoustic receivers in False Bay, South Africa. Within the Bay receivers were categorized as either Island (Seal Island) or Inshore (within 2 km of the shoreline). The insert shows the location of False Bay within the Western Cape region of South Africa. Satellite imagery: GoogleEarth. Date accessed: 07 09 2012. Co-ordinates: 34.216812 18.684759. Reprinted from Esri, DeLorme under a CC BY license, with permission from the Esri.

This study was conducted in False Bay, on the south-western tip of South Africa (34°04` - 34°23`S, 18°26` - 18°51`E) (Fig. 1). False Bay is the largest bay in southern Africa, with a total surface area of 1082 km^2^ and is over 30 km across at its widest point [36]. The coastline of False Bay forms part of the City of Cape Town metropole, which has a population of approximately 3.8 million people. The inshore region of the Bay is characterized by a broad range of habitats, including reef, sand and mixed reef and sand and supports a rich diversity of both teleosts and elasmobranchs [37], [38]. A single island (Seal Island) is located within the northern section of the Bay and is home to the second largest island-based breeding colony of Cape fur seals in South Africa (unpublished data). The population of seals varies from approximately 36 000 to 80 000 in the non-breeding and breeding season, respectively.

False Bay opens to the Atlantic Ocean, but is situated in an area of overlap between the cold Benguela Current in the west and warmer Agulhas Current to the south and east. False Bay falls within the warm-temperate marine bioregion, as described by Griffiths *et al*. [39] and experiences a Mediterranean climate with warm, dry and windy summers and cool, wet winters [38]. Water temperature in the Bay varies seasonally from a mean summer temperature of 21.5°C to a mean winter temperature of 13.2°C [38].

### Tagging of Sharks

White sharks were tagged at both Seal Island and the inshore area closest (6 km distance) to the island known as Strandfontein beach (Fig. 1). At Seal Island white sharks were attracted to the research vessel for tagging purposes using a standardized chumming and baiting method [25]. By contrast, on the inshore region tagging was achieved by actively searching for sharks at or near the water surface and then approaching them cautiously with the research vessel. We used a dense foam seal decoy, or a tuna head tied to a rope, to lure sharks to the research vessel. The size of the tagged shark was estimated to the nearest 0.5 m using the width of the research vessel (2.6 m) as a reference. The sex of the shark was determined by visual inspection for the presence or absence of claspers. Acoustic transmitters were deployed into the base of the first dorsal fin using a modified spear gun. Sharks were tagged with V16-5H-R04K (Code intervals: 150 to 300 s, 17×95 mm, battery life *ca*. 36 months) acoustic transmitters (Vemco Ltd. V16, Nova Scotia, Canada). Transmitters were encased in the manufacturer’s ‘shark case’ for added protection against damage. These transmitters periodically emit a pulse train of closely spaced 69 kHz pings, which serve to uniquely identify each shark. Each successfully decoded pulse train is recorded as a single detection by a VR2 receiver and stored in the receiver memory as the unique transmitter number, with date and time of detection [40].

### Acoustic Monitoring System

An array of 33 VR2 acoustic receivers (VEMCO Ltd.) was deployed in False Bay, South Africa during the period 1 April 2004 to 31 December 2010 to monitor the presence of white sharks. The array was arranged to ensure optimal coverage of both Seal Island [25] and the inshore regions of the Bay coast stretching from Cape Hangklip to Cape Point (Fig. 1). The inshore sites were chosen using two criteria, namely sites where white shark-human interactions had been previously recorded [41] and sites for which no information was available, but that were continuous along the inshore region of False Bay. Most inshore sites received two receivers, with the first receiver an average of 660 m from the shore (range 230–1230 m) and the second receiver an average of 1163 m meters (range 500–2260 m) from the shore along a straight line perpendicular to the coast. This design maximised the probability of shark detection in the inshore region of False Bay.

White sharks tagged in this study were also detected by acoustic receivers outside of False Bay in use by other researchers at three coastal regions off South Africa including, Gansbaai, Mossel Bay and Algoa Bay. These receivers were important in confirming that tags were still active and therefore being able to determine the number of tags at liberty each month, and that periods of ‘no detection’ in False Bay were not therefore a result of tag failure.

For the purposes of this study acoustic data were analyzed from 30 of these receivers deployed for the period 1 May 2005 to the 31 December 2007 (Fig. 1). Underwater receivers are omni-directional with a single channel (69 kHz) that listens continuously for the presence of coded-pulse acoustic transmitters [42]. Acoustic receivers were attached via a metal pole attached to concrete moorings deployed on the seafloor. Data from the VR2 receivers were downloaded with the VUE software provided by Vemco Ltd. Files were adjusted to account for time drift on the internal clocks and data were archived in an Access database.

### Data Analyses

For analyses of habitat use within False Bay, receiver sites were categorized into two regions 1) Seal Island (Island) and 2) the inshore region (Inshore). Sharks were split into one of two size categories, ≤3 or >3 m. The size categories were selected based on previous findings [34], [35] that white sharks ≤3 m feed predominantly on teleosts and elasmobranchs, while those >3 m supplement their diet with marine mammals like seals. We thus predicted differences in habitat use between these two size categories. Statistical tests were performed using Stata software (version 11; StataCorp).

#### Residency

The number of days individual tagged sharks were monitored over the study period (date from first tagged, to date of last acoustic detection) was determined and referred to as the ‘monitoring period’. Residency of all tagged sharks was assessed on a daily basis, with individuals considered present in the study area if more than one detection was recorded on any receiver in the array on a given day *sensu* Carlson *et al*. [43]. The number of days that each individual was present in False Bay over the study period was plotted on a timeline and categorized as ‘days detected’. We evaluated whether sex or size influenced white shark residency in False Bay using *t*-tests to compare the 1) tag duration (in days from date of tagging in False Bay to date last detected on any receiver along the South African coast), and 2) the number of days detected in False Bay for male and female and then for sharks ≤3 and >3 m, respectively.

#### Generalized linear mixed models

These analyses were based on the number of visits of each shark to the two regions of False Bay (Inshore vs. Island). A single visit to either the Island or Inshore was defined as a recording of a tag at any single receiver within that region followed by a period of at least 30 minutes during which that tag was not detected by any other receiver within that region. The numbers of visits were averaged per month to explore the seasonal visitation patterns for males and females, for both size categories (≤3 and >3 m), across years and for sharks tagged at Island vs. Inshore. To investigate the effects of season, sex and shark size on habitat use we used a generalized linear mixed effect model (GLMM) [44] with a binary response defined by sharks present on the Inshore (0) or sharks present at the Island (1). Generalized linear models accommodate different (non-normal) response types, by allowing for the generalization of ordinary regression techniques. In this instance, since the response was binary, a logistic model was used. The model included shark-specific random effects, which accounted for the variation in movement patterns by individual sharks.

The model was defined as follows:
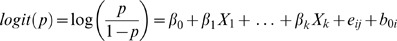



Where 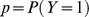, 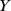 is the response variable, 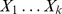 are the 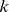 explanatory variables, and 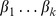, the 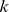 corresponding coefficients, is the shark-specific random intercept effect, and where 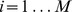 sharks, and 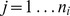 observations on each shark.

The recordings were categorized into season (where summer represented December - February, autumn March-May, winter June-August and spring September-November) (variable SEASON). Sex of the sharks (variable SEX) and size of the shark (variable SIZE) were also indicated. The year of study (variable YEAR), and whether the shark was tagged at the Island or Inshore (variable AREA TAGGED) were also considered for inclusion in the model. A description of the independent variables used in the GLMM analysis are provided in Table 1. The impacts of the various explanatory variables were assessed by interpreting the odds ratios, which were obtained by exponentiating the relevant beta coefficients.

**Table 1:** Summary of the independent variables used in the GLMM analysis.

Independent variable	Type	Description	Values
SEASON	Categorical	Identifies recordings made during seasons	Summer (Dec, Jan, Feb) Autumn (Mar, Apr, May) Winter (Jun, Jul, Aug) Spring (Sep, Oct, Nov)
SEX	Categorical	Sex of the shark	Female; Male
SIZE	Categorical	Size of the shark	≤3 m; >3 m
AREA TAGGED	Categorical	Area where sharks were tagged	Inshore; Island
YEAR	Categorical	Identifies which year recordings made	2005, 2006, 2007

The response term indicated the presence of a shark at either the Inshore region or at the Island.

Model building followed an all subsets procedure, but was led by specific hypotheses. We compared models and selected the best-fitting model by using standard selection criteria (AIC and BIC) to determine which variables best explained the variability in the data [45]. The BIC adjusts for the number of observations and variables in the model, and so will not decrease if the variable added to the model in the latest step does not sufficiently improve the fit, i.e. if its inclusion is not justified. Its use thus allowed us to penalize for non-parsimonious models. Likelihood ratio tests were also used to determine whether the inclusion of additional variables in the model significantly improved the amount of variability explained. In all instances we were looking for the best fitting predictive model i.e. the model that both fits the data and is most simple. Finally, we checked that the assumptions of the model were met by examining residual and random effects diagnostic plots.

The issue of pseudo-replication was managed by including Shark-ID as a random effect. The error structure of GLMM corrects for the non-independence of statistical units due to shared temporal structure, and permits the ‘random effects’ variance explained at different levels of clustering to be decomposed. The inclusion of individual shark as a random effect enabled us to account for lack of independence between observations within each identified shark.

## Results

### Sex and Size of Tagged Sharks

A total of 53 white sharks were tagged with acoustic transmitters in False Bay between 1 May 2005 and 31 December 2007 (2005, n = 23; 2006, n = 25; 2007, n = 5). Additionally three sharks tagged in 2004 at Seal Island as part of a long-term study, returned in 2005 and were included in the analysis, bringing the total number of acoustically monitored sharks for the study period to 56 (Table 2). Tagging took place predominantly at Seal Island (45 out of 56 individuals or 80%) compared to the Inshore region (11 out of 56 or 20%) (Table 2). Inshore tagging was only conducted during the summer of 2006/2007 and only female sharks were encountered in the eleven tagging sessions. Sharks fell predominantly into the >3 m category (40 of 56 or 71%) and were mostly female (39 of 56 sharks or 69.1%). Tagged animals in this study (based on their estimated size) likely represent mostly juveniles and sub-adults.

**Table 2:** Summary of tag deployments on white sharks *Carcharodon carcharias* in False Bay between May 2005 and December 2007.

Shark ID	TL (cm)	Size Category	Sex	Area Tagged	Date Tagged	Date of last acoustic detection in False Bay	Tag duration (days)	No. of days detected in False Bay
28	300	≤3	F	Island	09/03/04	08/31/05	363	22
520	400	>3	M	Island	04/25/04	08/09/05	472	69
521	370	>3	F	Island	04/25/04	06/13/05	415	103
533	340	>3	F	Island	04/06/06	06/15/06	71	44
534	330	>3	M	Island	04/06/06	08/06/06	123	88
545	280	≤3	F	Inshore	11/14/06	12/31/07	413	180
546	280	≤3	F	Island	04/28/06	12/27/06	244	210
547	350	>3	F	Island	06/30/06	07/27/07	393	282
548	320	>3	F	Island	04/28/06	11/04/07	556	164
549	300	≤3	F	Island	08/17/06	07/21/07	339	185
551	320	>3	F	Inshore	11/14/06	11/06/07	358	149
552	250	≤3	M	Island	06/30/06	07/13/06	14	10
553	340	>3	M	Island	06/30/06	08/01/07	398	110
554	340	>3	M	Island	07/03/06	08/18/06	47	33
556	380	>3	F	Island	08/09/06	10/30/06	83	76
557	280	≤3	M	Island	08/17/06	10/24/06	69	40
558	370	>3	F	Inshore	10/06/06	02/27/07	145	110
560	170	≤3	F	Inshore	11/13/06	04/17/07	156	129
562	340	>3	F	Inshore	11/14/06	05/23/07	191	181
601	450	>3	F	Island	08/25/05	09/16/05	23	21
603	380	>3	F	Island	05/20/05	01/03/06	229	169
604	350	>3	M	Island	08/29/05	09/21/06	389	164
605	320	>3	M	Island	08/24/05	11/07/06	441	67
606	350	>3	F	Island	06/04/05	06/10/05	7	4
608	360	>3	F	Island	06/04/05	10/03/05	122	84
609	360	>3	M	Island	06/04/05	08/19/05	77	64
610	420	>3	F	Island	06/04/06	06/23/06	20	7
611	250	≤3	F	Island	09/02/05	05/07/06	248	151
612	220	≤3	M	Island	05/19/06	06/15/07	393	74
613	320	>3	M	Island	06/28/05	09/16/06	446	71
614	360	>3	F	Island	06/06/05	07/23/05	48	28
615	320	>3	M	Island	08/30/05	06/18/06	293	22
616	350	>3	M	Island	06/06/05	06/14/05	9	9
617	380	>3	F	Island	06/06/05	08/30/05	86	39
618	400	>3	F	Island	06/16/05	06/24/05	9	9
619	350	>3	M	Island	06/16/05	08/10/06	421	88
620	360	>3	F	Island	06/17/05	12/27/05	194	180
621	300	≤3	F	Island	06/06/05	11/17/05	165	126
622	340	>3	M	Island	06/10/05	10/18/05	131	60
623	330	>3	F	Island	06/10/05	01/22/06	227	60
624	500	>3	F	Island	06/06/05	06/10/05	5	5
625	300	≤3	F	Island	06/10/05	08/01/05	53	16
626	250	≤3	F	Island	06/10/05	01/08/06	213	149
627	350	>3	M	Island	04/20/06	04/23/06	4	2
628	330	>3	M	Island	05/21/06	06/08/06	19	17
630	340	>3	F	Island	05/25/06	09/29/06	128	65
632	300	≤3	F	Inshore	11/13/06	01/28/07	77	61
633	330	>3	F	Inshore	01/26/07	11/08/07	287	257
634	380	>3	F	Inshore	11/14/06	04/17/07	155	135
635	300	≤3	F	Inshore	11/14/06	12/29/06	46	37
636	300	≤3	F	Inshore	11/14/06	11/24/06	11	11
637	400	>3	F	Inshore	01/17/07	08/08/07	204	147
638	400	>3	F	Island	03/10/07	11/02/07	238	169
639	300	≤3	F	Island	06/12/07	12/31/07	203	179
642	340	>3	F	Island	09/14/07	11/03/07	51	47
602607	350	>3	F	Island	06/17/05	01/14/06	212	153

Data include the Shark ID number, total length (TL) (estimated to nearest 0.5 m), size category, sex, area tagged, date of tagging, the last date the tag was recorded in False Bay, the tag duration (days) and the total number of days detected in False Bay.

### Movement Rate between Sites

100% of tagged males (n = 16) and females (n = 30) were detected at the Island during winter, with similar high levels of detection on the Inshore for both sexes (94% and 97% respectively). Female detection rates remained high at both the Island (82%, n = 22) and the Inshore (95%) during summer, whilst male detection was lower at both the Island (22%, n = 9) and the Inshore (11%).

### Residency

**Figure 2:**
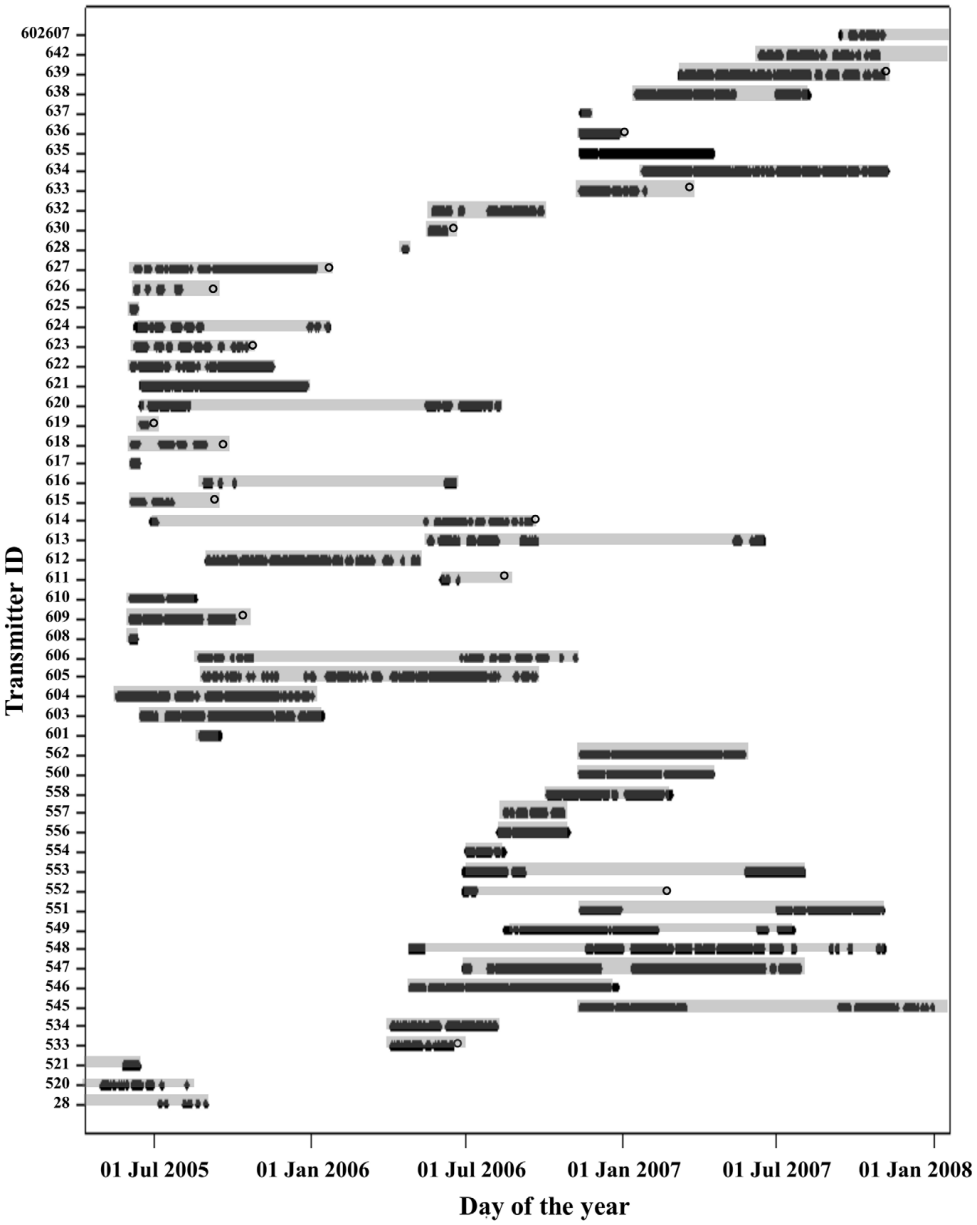
Timeline of the daily detections of acoustic tagged individual sharks in False Bay from 1 May 2005 to 31 December 2007. The first point indicates the date the shark was tagged, time at liberty is represented by grey bars and an open circle indicates the last detection as being on a receiver outside of False Bay, at Gansbaai, Mossel Bay or Algoa Bay.

Tagged white sharks were monitored on the acoustic array for 975 days and detection patterns varied among individuals (Fig. 2). Tag duration ranged from 2–556 days (median = 160.5 days) and the number of days detected ranged from 2–282 days (median = 72.5 days). The average tag duration for males was 220.35 days (±45 days) and for females was 179.18 days (±21 days). The average tag duration for sharks ≤3 m was 187.94 days (±34 days) and for sharks >3 m was 193.18 days (±25 days). There were no significant differences between the tag duration between males and females (t = 0.92, df = 54, p = 0.8203), or between sharks in the two size categories ≤3 and >3 m (t = 0.1146, df = 54, p = 4546). The average number of days males and females were detected in False Bay was 58.12 days (±10 days) and 106.26 days (±12 days) respectively. The average number of days sharks ≤3 m were detected was 98.75 days (±17 days) with sharks >3 m being detected an average of 88.8 days (±11 days). The number of days females were detected in False Bay was significantly more than males (t = 2.46, df = 54, p = 0.0086), but there was no significant difference in the number of days detected between the two size categories (t = 0.47, df = 54, p = 0.6816).

### The Effects of Season, Size and Sex on Shark Presence within False Bay, at Seal Island and the Inshore Region

GLMM were used to examine the influence of season, sex and size on white shark presence at Seal Island versus the Inshore. Table 3 shows the various stages of the model building procedure. Variables were initially considered independently of one another (Stage I). Of these initial models, the model including season was selected as the best (assessed using AIC and BIC as described). Stages II and III built on the initial model, with each additional explanatory variable considered in turn. Finally, in Stage IV, interaction terms were considered. The likelihood ratio test was used to determine whether the best model at each successive stage was significantly better than the previous best model. The final model included season, sex, and an interaction term between season and sex (Table 3).

**Table 3:** Generalized linear mixed models constructed for predicting whether white sharks *Carcharodon carcharias* would be present at the Island versus Inshore.

	Model Description	AIC	BIC	Lrtest	Lrtest p-value
Stage I	1) Sex	17658.55	17681.87	NA	NA
	2) Season	11974.6	12013.46	NA	NA
	3) Size	17669.11	17692.43	NA	NA
	4) Area Tagged	17655.29	17678.61	NA	NA
Stage II	5) Season+Sex	11960.15	12006.79	5 vs. 2	16.45 (<0.0001)
	6) Season+Size	11974.79	12021.43	NA	NA
	7) Season+ Area Tagged	11968.96	12015.6	NA	NA
Stage III	8) Season+Sex+Size	11961.24	12015.65	8 vs. 5	0.9 (0.339)
	9) Season+Sex+Area tagged	11959.02	12013.43	9 vs. 5	3.13 (0.0767)
Stage IV	10) Season+Sex+Season:Sex	11865.59	11935.54	10 vs. 5	100.56 (<0.0001)

The best-fitting model was selected by using standard selection criteria (AIC and BIC) to determine which variables best explained the variability in the data and likelihood ratio tests were used to determine whether the inclusion of additional variables in the model significantly improved the amount of variability explained.

Due to tagging only taking place on the Inshore during the summer of 2006, the area tagged and year was confounded, and thus the effects of year (and its interaction with season), were considered only at the end of the model building procedure. Small effects of year were observed, however, since the inclusion of year and its interaction with season would overcomplicate the model, and since the “yearly” differences are assumed to be more related to tagging times, and finally, since year is not of any primary interest, and the same trends are observed in all three years of observation (both seasonal and sex and the interaction of the two), as per the model accounting for year (Table S1), the simpler model (without year) is presented in detail here.

A summary of the results from the final model (excluding year) is provided in Table 4. For each season and sex combination, the likelihood of a white shark visit occurring at the Island versus Inshore is described, using predicted odds ratios and their corresponding 95% confidence intervals. Odds ratios that are greater than 1 indicate an increased likelihood of an Island visit; whilst those that are less than 1 indicate a decreased likelihood. All odds ratios were statistically significant, with the exception of that for males in spring. The model results indicate that there is a marked seasonal effect, and that this effect differs depending on the sex of the shark. For males, Island visits are more likely year round, with a peak likelihood in the winter months (males are 32 times more likely to be seen at the Island than Inshore). However, female visits to the Island are less likely than Inshore visits in summer and spring. The large amount of variability observed for the males may be explained by the scarcity of observed visits to either region (Island or Inshore) in the summer and spring months: the only observed movements in these months are for a single shark, frequenting the Island.

**Table 4:** Results from the Generalized Linear Mixed Effects Model (GLMM) showing the likelihood of white sharks *Carcharodon carcharias* being at the Island versus Inshore across seasons.

Season	Males	Females
Summer	12.86 (4.19, 39.51)	0.06 (0.04, 0.09)
Autumn	10.77 (4.9, 23.64)	2.35 (1.47, 3.77)
Winter	32.37 (15.44, 67.90)	6.73 (4.23, 10.68)
Spring	1.89 (0.87, 4.12)	0.17 (0.10, 0.27)

**Figure 3:**
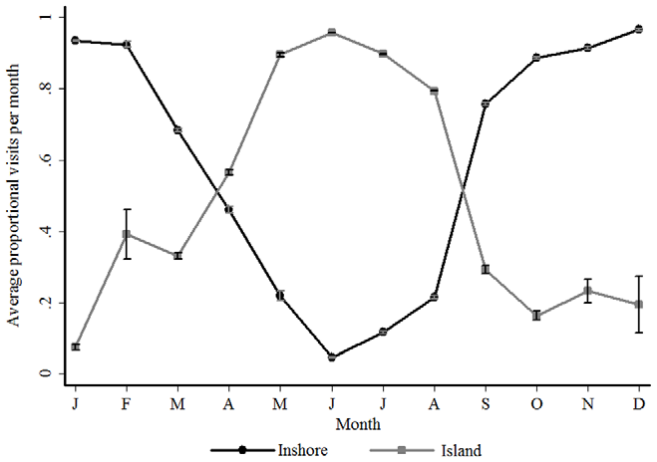
The proportion of visits to each region in the Bay for all years combined. Average (± s. d.) proportion of visits to the Inshore (black line) and Island (gray line) areas of False Bay for each month of the year.

**Figure 4:**
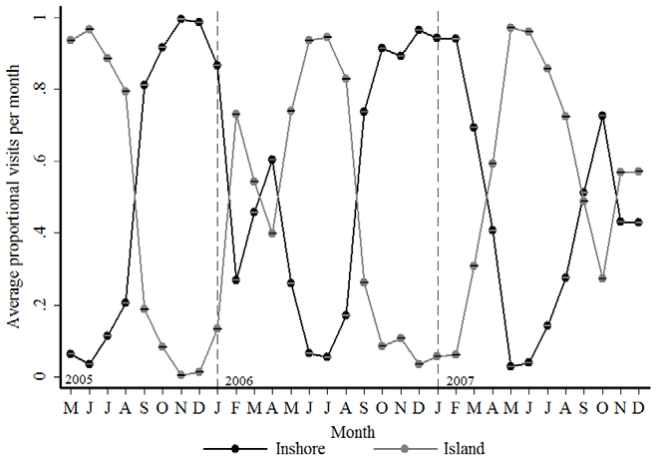
The proportion of visits to each region in the Bay for all years. Average (± s. d.) proportion of visits to the Inshore area (black line) and Seal Island (grey line) for tagged male and female sharks for each month of the year (Jan-Dec) from May 2005 to December 2007.

Figure 3 shows the proportion of visits to the Inshore and Island regions in each month for all sharks. There is a clear seasonal pattern, with peaks in visits around the Island in the autumn and winter months (April - August), and at the Inshore region during spring and summer (September - March). This trend was consistent irrespective of the year (Fig. 4).

**Figure 5:**
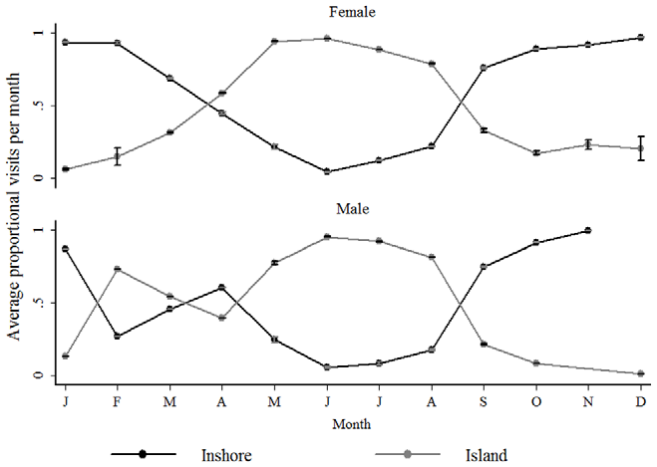
The proportion of visits to each region in the Bay by sex. Average (± s. d.) proportion of visits to the Inshore area (black line) and Island (grey line) for tagged male and female sharks for each month of the year (Jan-Dec) from May 2005 to December 2007.

A comparison of the proportion of visits per region in each month, over the years for each sex, (Fig. 5) reveals that while both sexes exhibit a clear peak in visits to the Island during the winter months only females exhibit a seasonal peak (summer) on the Inshore region. Males were seldom detected in the Inshore region in any month and were also rarely detected in summer at either the Island or Inshore region.

**Figure 6:**
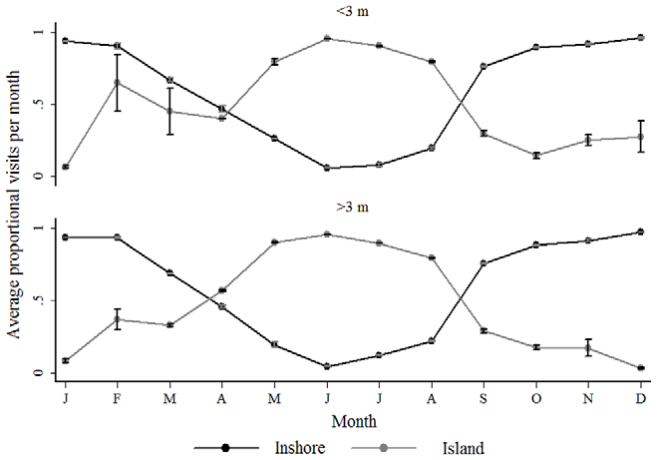
The proportion of visits to each region in the Bay by size. Average (± s. d.) proportion of visits to the Inshore area (black line) and Seal Island (grey line) for tagged < = 3 and >3 m sharks for each month of the year (Jan-Dec) from May 2005 to December 2007.

The trend of aggregating around the Island during winter and Inshore during summer was not influenced by the size of the shark (Fig. 6).

## Discussion

Tagged white sharks were detected in False Bay in all months of the year and across all years, with predictable seasonal aggregations in two distinct regions within the Bay. This suggests both a high level of residency and a strong annual rhythm of habitat use for this coastal region. White shark use of the Bay varied significantly with both the season and the sex of the shark, but not with shark size. In autumn and winter both males and females of different sizes aggregated at the Cape fur seal colony (Island), where they were observed to feed predominantly on young of the year seals. In the spring and summer months there was marked sexual segregation, with females frequenting the Inshore areas and males seldom being detected at any acoustic stations in the Bay. Out of eleven field trips to tag sharks on the Inshore over the 2006/2007 summer season, only female sharks were encountered and thus tagged, which further strengthens our observations.

White shark aggregations at pinniped rookeries are well-established and almost unanimously considered to reflect convergence of predators on a seasonally abundant, high quality food resource [46], [47], [48], [21], [22], [23], [24], [25], [15]. Seal Island, False Bay is a known white shark aggregation site and the convergence of sharks over autumn and winter are generally attributed to the seasonal increase in the abundance of predator-naïve seals [25], [49]. However, this study further identified the Inshore region of False Bay as another important and frequently-used region by female white sharks ranging in size from 1.7–5 m. Few studies have looked at habitat use along Inshore areas not associated with seal colonies. Recently Bruce *et al.* [15] reported temporary seasonal residency of newborn and juvenile sharks near beaches in eastern Australia, identifying two primary residency sites (periods of residency at these two sites ranged from 21–122 days) along a coastal stretch of 2000 km. This is similar to our finding of temporary (seasonal) residency in the Inshore habitat, but differs in being sex-biased rather than size-biased.

The causes of white shark aggregations and the marked sexual segregation found in the Inshore region of False Bay are not known. Habitat segregation by sex appears common among sharks [50], [51], where adult males and females within a species use different habitats either within the same or different areas [52]. Habitats may be selected differentially by the sexes for social, thermal or forage-related reasons, for example see [51]. This behaviour can result in either females or males being more or less susceptible to threats [52]. While we have no data for social interactions between white sharks in False Bay there is information available on both thermal and food variables within the Bay that may help explain the marked seasonal patterns in aggregation sites. Water temperature within the Bay is highest in spring and summer and is associated with inshore diatom blooms, which promote spawning and recruitment by a diverse assemblage of fish [53], [37], [38] in addition to higher fish abundance [37], [38]. Given the close association between prey abundance and shark distribution [54], [55], [56]; [57], [58] it is possible that the combination of increased difficulty in catching juvenile seals at the island, with concomitant increase in the availability of a variety of fish species in the Inshore region, may explain the marked seasonal shift in white shark habitat use within the Bay.

The diet of white sharks on the Inshore areas of False Bay is unknown, but they have been observed feeding on seasonally abundant fish such as white steenbras (*Lithognathus lithognathus*), yellowtail (*Seriola lalandi*) and depredating on various elasmobranch species frequently caught by fishermen in False Bay (unpublished data). It is therefore reasonable to expect white sharks to move towards and forage on seasonally abundant prey resources, as occurs in other fishes in False Bay, or similar to other large predators in other systems [58], [59]. However, this seasonal shift in prey abundance from the Island to the Inshore does not explain male movement patterns within False Bay, with the males seldom being detected Inshore and outside of the winter months. We hypothesize that the males leave the Bay and disperse along the Southern African coast during spring and summer. Current satellite tracking data provides strong support for this hypothesis, but it remains to be verified through appropriate analyses (unpublished data).

In white sharks, sex-specific seasonal visitation patterns have been identified at select aggregation sites, where males and females arrive and depart at different times. In central California sex-specific visitation patterns at aggregation sites are thought to be linked to the 12–18 month gestation period of females, who only visit every second year, whilst males return annually [31]. A similar pattern has been observed at Guadalupe Island, Mexico and has also been attributed to the sex-specific differences in the reproductive cycle [24]. At the Neptune Islands, Australia shark occurrence is biased towards males and more males are observed in months with cooler water temperatures and more females in months with warmer water temperatures, giving rise to the hypothesis that the segregation is related to water temperature [32], [60]. In these studies it has been proposed that warmer waters may facilitate optimum growth of developing embryos (unlikely in our study as most sharks were immature), but has also been suggested to increase growth rates and so enable females to achieve maturity size at a similar age to males [50], [61]. Conversely, is has been suggested that male sharks may select cooler waters for optimal sperm production [62]. Our findings are similar to those reported for sevengill sharks (*Notorynchus cepedianus*) in Tasmania, where males and females are present at coastal sites during summer, but during winter, males moved out of the coastal areas migrating north, while females remained at the coastal site [63].

Our study provides the first evidence of sexual segregation at a fine spatial scale and demonstrates that sexual segregation in white sharks is not restricted to adults, but is apparent for juveniles and sub-adults too. We found no evidence of sexual segregation at Seal Island, with individuals of both sexes and a range of sizes aggregating here each winter. Our findings strongly suggest that both the Island and Inshore region of False Bay should be classified as critical areas for the conservation of white sharks in South Africa and globally. Currently no critical area conservation plans exist for either False Bay, or anywhere in South Africa. Females are particularly at risk, due to their frequent use of the Inshore areas of the Bay, which are impacted by fishing, pollution, and damage to natural habitat from coastal development. Furthermore, the peak in female use of the Inshore region in the summer months corresponds with the annual recreational peak for this zone [64]. Shark attacks (on average one per year in False Bay since 1960) put tremendous pressure on local conservation and management authorities to mitigate these events and there are frequent calls for the removal of sharks e.g. culling using drum lines or gill nets or their exclusion from the more popular Inshore recreational areas e.g. barriers or exclusion nets. A thorough understanding of how sharks are utilizing False Bay will enable managers and conservation authorities to better educate recreational users of the Bay, in addition to allocating resources to mitigate potential conflict (e.g. shark spotter programme) during the high-risk periods.

## Supporting Information

Table S1 **Results from the Generalized Linear Mixed Effects Model (GLMM) (with year) showing the likelihood of white sharks being at the Island versus Inshore.**
(DOCX)
