# Supplementary material for: Residency, Habitat Use and Sexual Segregation of White Sharks, Carcharodon carcharias in False Bay, South Africa
Source: PLoS One. 2013 Jan 28;8(1):e55048. doi: 10.1371/journal.pone.0055048 (PMC3557240; doi:10.1371/journal.pone.0055048)
Supplement: Table S1 — Results from the Generalized Linear Mixed Effects Model (GLMM) (with year) showing the likelihood of white sharks being at the Island versus Inshore. (DOCX) [file pone.0055048.s001.docx]

| Year | Season | Males | Females |
| --- | --- | --- | --- |
| 2005 | Summer | 1.52 (0.31, 7.40) | 0.02 (0.01, 0.07) |
|  | Autumn | 74.31 (22.02, 250.82) | 15.27 (5.89, 39.61) |
|  | Winter | 29.11 (12.70, 66.72) | 13.10, (7.31, 23.48) |
|  | Spring | 1.15 (0.51, 2.57) | 0.17, (0.09, 0.30) |
| 2006 | Summer | 11.77 (3.77, 36.71) | 0.03 (0.02, 0.06) |
|  | Autumn | 8.46 (3.85, 18.63) | 0.33, (0.19, 0.59) |
|  | Winter | 31.48 (14.84, 66.78) | 2.71 (1.57, 4.68) |
|  | Spring | 2.86 (1.24, 6.59) | 0.08 (0.05, 0.14) |
| 2007 | Summer | - | 0.06 (0.03, 0.10) |
|  | Autumn | 196.53 (36.75, 1051.07) | 2.52 (1.48, 4.29) |
|  | Winter | 179.41 (34.86, 923.46) | 5.04 (2.93, 8.65) |
|  | Spring | - | 0.35 (0.20, 0.62) |

Table S1. Results from the Generalized Linear Mixed Effects Model (GLMM) (with year) showing the likelihood of white sharks being at the Island versus Inshore
